# WISP-1 induced by mechanical stress contributes to fibrosis and hypertrophy of the ligamentum flavum through Hedgehog-Gli1 signaling

**DOI:** 10.1038/s12276-021-00636-5

**Published:** 2021-06-22

**Authors:** Chao Sun, Qinghong Ma, Jian Yin, Han Zhang, Xinhui Liu

**Affiliations:** Department of Spine Surgery, The Affiliated Jiangning Hospital with Nanjing Medical University, Nanjing, Jiangsu 211100 China

**Keywords:** Ageing, Trauma, Bone

## Abstract

Ongoing chronic fibrosis and hypertrophy of the ligamentum flavum (LF) is an important cause of lumbar spinal canal stenosis (LSCS). Our previous work showed that WNT1-inducible signaling pathway protein 1 (WISP-1) is a critical driver of LF fibrosis. However, the potential mechanism has not been explored. Here, we found that Gli1 was upregulated in hypertrophic LF tissues and required for fibrogenesis in fibroblasts. Moreover, mechanical stretching increased the expression of WISP-1 in LF fibroblasts. Furthermore, WISP-1 induced fibrogenesis in vitro through the Hedgehog-Gli1 pathway. This conclusion was supported by the fact that WISP-1 activated the Hedgehog-Gli1 pathway in LF fibroblasts and that cyclopamine attenuated the effect of WISP-1-induced fibrogenesis. WISP-1 also promoted the transition of fibroblasts into myofibroblasts via the Hedgehog pathway. Importantly, a hypertrophic LF rabbit model induced by mechanical stress also showed pathological changes in fibrosis and elevated expression of WISP-1, Gli1, and α-SMA. Therapeutic administration of cyclopamine reduced collagen expression, fibroblast proliferation, and myofibroblast differentiation and ameliorated fibrosis in the mechanical stress-induced rabbit model. Collectively, our findings show mechanical stress/WISP-1/Hedgehog signaling as a new fibrotic axis contributing to LF hypertrophy and identify Hedgehog signaling as a therapeutic target for the prevention and treatment of LF fibrosis.

## Back pain: stopping signaling could soothe sore spine

A signaling pathway that triggers fibrosis and abnormal growth (hypertrophy) of ligaments in the spine could be targeted to treat an age-related lumbar condition. In lumbar spinal canal stenosis, the ligamenta flava (LF), literally the ‘yellow ligaments’ between vertebrae, expand due to hypertrophy and fibrosis. This narrows the canal carrying the spinal cord, leaving sufferers in significant pain. Previously, Xinhui Liu and co-workers at the Affiliated Jiangning Hospital with Nanjing Medical University, China, demonstrated that the WISP-1 protein drives LF fibrosis and is highly expressed in hypertrophic LF. Now, the team have shown that expression levels of WISP-1 increase with mechnical stretching of LF cells over time. WISP-1 then activates a signaling pathway that promotes LF cell proliferation and the differentiation of fibroblast cells, inducing fibrosis. Inhibiting this signaling pathway could partially prevent the progression of stress-induced LF fibrosis.

## Introduction

Lumbar spinal canal stenosis (LSCS) occurs mainly in the elderly population with symptoms of back and/or leg pain, numbness, and intermittent claudication.^1^ The development of LSCS is attributable to several pathogenic factors, such as disc protrusion, facet joint degeneration, and ligamentum flavum (LF) hypertrophy. LF hypertrophy is considered an important factor in the development of LSCS.^2^

Anatomically, the LF covers the posterior part of the dural sac, acting to limit the range of intervertebral movement and protect the spinal cord.^3,4^ The normal LF is an elastic structure rich in elastic fibers. In contrast, the hypertrophic LF shows fibrotic changes characterized by a loss of elastic fibers and an increase in collagen fiber content.^5,6^ Fibrosis is a very common pathophysiological change in many chronic diseases.^7^ Fibrosis is also the main pathological feature of LF hypertrophy.^5,8^ However, the detailed pathological mechanism of LF fibrosis is not clear.

The CCN family is composed of six matrix proteins that function mainly in extracellular matrix (ECM) regulation.^9–11^ Among them, WISP-1, also called CCN4, has been studied the most extensively and is involved in fibrotic processes in a variety of organ systems.^12,13^ Previous work by our group showed that WISP-1 is highly expressed in hypertrophied LF tissue and is closely associated with LF fibrosis.^14^ However, little is known about the mechanism by which WISP-1 is activated and the downstream mechanism of WISP-1 in driving fibroblast activation in the context of LF fibrosis. The results presented in this study not only deepen the understanding of the role and mechanism of WISP-1 in LF fibrosis but also offer a theoretical basis for developing new drugs for LF hypertrophy.

## Materials and methods

### Subjects

The present study was approved by the Ethics Committee of Nanjing Medical University. From April 2018 to July 2019, 21 patients with degenerative LSCS who underwent posterior decompressive laminectomy were recruited for this study. The diagnosis of LSCS was based on both clinical symptoms and radiological examinations. The inclusion criteria were as follows: age between 55 and 70 years, L4/5 level stenosis, and LF hypertrophy confirmed by magnetic resonance imaging (MRI). Patients with comorbidities such as spinal tuberculosis, scoliosis, and skeletal dysplasia were excluded. The nonhypertrophied LF samples used in the study were obtained from age-matched patients with lumbar disc herniation (LDH) who underwent posterior laminectomy. MRI was used to confirm that the LF was nonhypertrophic in this group. Informed consent was obtained from every patient prior to enrollment in this study. The surgery was performed by three experienced spinal surgery specialists in the authors’ hospital. The detailed characteristics of the patients involved in this study are summarized in Table 1.

**Table 1:** Comparison of data between the two groups.

Index	LSCS group	LDH group	*P* value
Number of patients	21	21	
Mean age (years)	61.3 ± 4.9	59.0 ± 4.4	<0.05
Sex (male/female)	10/11	12/9	
Level	L4/5	L4/5	
LF thickness (mm)	4.75 ± 0.39	2.57 ± 0.69	<0.05
Fibrosis score	3.14 ± 0.85	0.95 ± 0.92	<0.05

### LF thickness measurement and fibrosis evaluation

**Fig. 1:**
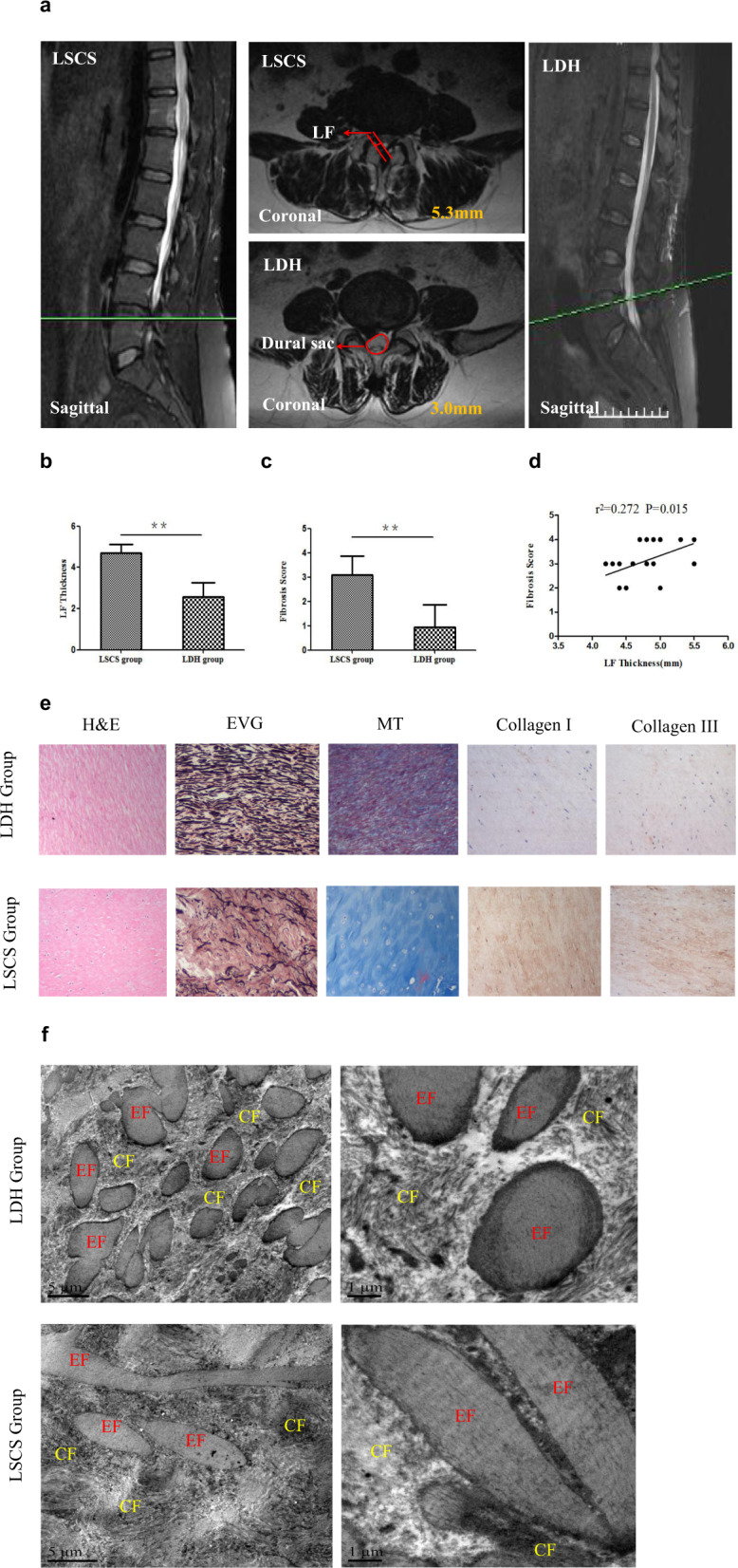
MRI of the lumbar spine and morphological and structural observations of LF tissues. **a** Observation of LF tissue on coronal and sagittal T2-weighted MRI. The LF thickness was measured at the facet joint level on T2-weighted MRI. **b**, **c** Comparison of the LF thickness and fibrosis score between the two groups. **d** Correlation analysis of the LF thickness and fibrosis score in the LSCS group. **e** Representative images of H&E, EVG, MT (*n* = 21) and collagen staining of LF specimens (*n* = 3). Scale bar, 100 µm. **f** TEM of the ultrastructure of the LF. Scale bar, 5 µm. EF elastic fibers, CF collagen fibers, MT Masson’s trichrome, LF ligamentum flavum, LSCS lumbar spinal canal stenosis, LDH lumbar disc herniation. **P < 0.01.

According to a previously described method,^15^ LF thickness was assessed at the facet joint level on T2-weighted MRI for all 42 patients. The measurement was performed using Picture Archiving and Communication Systems (PACS) software (Nanjing Medical University, Nanjing, China). The value was measured three times for each patient by one experienced spine surgeon, and the average value was considered the LF thickness (Fig. 1A).

All LF samples collected for this study were obtained only from the dorsal layer of the LF. The LF samples were fixed in 4% paraformaldehyde (PFA) phosphate buffer solution for 48 h and subsequently embedded in paraffin in preparation for histopathological analysis. The samples were sliced along the coronal plane for each specimen at the same level by using a paraffin microtome. After dewaxing, the sections were stained using hematoxylin and eosin (H&E) staining, Masson’s trichrome (MT) staining, and Elastica van Gieson (EVG) kits (JianChen, Nanjing, China). H&E and EVG staining were used to evaluate the morphology and structure of the LF. Moreover, the degree of LF fibrosis was assessed according to the results of MT staining. The fibrotic area was quantified with Image-Pro Plus 6.0 (IPP6.0). As previously described,^16^ the grade of LF fibrosis was classified on the basis of the percentage of collagen in the entire area of the sample, as follows: Grade 0, less than 20%; Grade 1, between 20 and 25%; Grade 2, between 25% and 50%; Grade 3, between 50% and 75%; and Grade 4, over 75%.

### Immunohistochemistry

The LF specimens were fixed in 10% neutral formalin, embedded in paraffin and finally cut into sections with a thickness of 4 μm. After dewaxing in xylene and rehydrating in a series of alcohol solutions, the sections were incubated with primary antibodies against Gli1, Sonic Hedgehog (Shh), collagen I, collagen III, and α-Smooth muscle actin (α-SMA) (Proteintech) at the optimum dilutions recommended by the manufacturers overnight at 4 °C. Subsequently, these sections were incubated with the respective secondary antibodies (Proteintech) at room temperature. Immunohistochemical analysis was performed, and the results were visualized by confocal laser scanning microscopy (Zeiss, Germany).

### Transmission electron microscopy (TEM)

After the LF specimens were harvested during the operation, they were immediately cut into small pieces of ≈0.8 mm^3^ and subsequently fixed in 2.5% glutaraldehyde for 3 h. Next, the samples were washed three times for 15 min per wash using phosphate-buffered saline (PBS). After dehydration with propylene oxide three times for 30 min each time, they were embedded in Epon and cut into ultrathin sections of 70 nm thickness using an ultramicrotome (EM UC7, Leica, Germany). Finally, the sections were stained with 2% uranyl acetate. The morphology and structure of the LF were analyzed under a transmission electron microscope (LVEM5, Delong America).

### LF cell culture

The LF specimens obtained from patients during surgery were washed three times with PBS (Gibco), minced into small fragments of ≈0.5 mm^3^ and digested using 0.2% type I collagenase (Gibco) at 37 °C for 1.5 h. Subsequently, the digested pieces were washed with Dulbecco’s modified Eagle’s medium (DMEM, Gibco) and cultured with DMEM supplemented with 10% fetal bovine serum (Sigma), 100 U/ml penicillin and 100 pg/ml streptomycin (Sigma) in a 5% CO_2_ humidified incubator at 37 °C. The cell culture medium was changed every 2–3 days. Second-generation cells were used for subsequent experiments.

The expression levels of collagen I and III were detected by immunostaining and observed under a confocal microscope (XTL3230-DIC, Shanghai, China) to identify the cell type. Second-passage LF cells were cultured and stimulated with different concentrations of recombinant human WISP-1 ranging from 0 to 150 ng/ml for 24 h. For Hedgehog signaling inhibition experiments, 10^5^ cells were seeded in each well of 6-well plates. Then, the indicated dose (5 μM) of inhibitor (cyclopamine, Sigma, USA), which did not exhibit cytotoxicity, was added to the culture medium, and the cells were incubated for 24 h. Finally, the cells were collected for subsequent experiments.

### Cell viability assays

LF cells were seeded in 96-well plates at 10^5^ cells per well and cultured at 37 °C under 5% CO_2_ for 24 h. Then, the LF cells were incubated in 80 µl of DMEM supplemented with 20 μl of 5 mg/ml 3-(4,5-dimethylthiazol-2-yl)−2,5-diphenyltetrazolium bromide (MTT) solution (Beyotime, Nanjing, China) for 4 h. After medium removal, the formazan crystals were dissolved in 100 μl of DMSO/well, and the absorbance of the solution was measured at 490 nm. For colony-forming unit detection, the cells were pretreated with Crystal Violet Staining Solution for 10 min at 37 °C. For EdU and BrdU detection, the cells were pretreated with EdU and BrdU working solutions (Beyotime, Nanjing, China) for 2 h at 37 °C. Finally, the cells were observed under a microscope (Olympus, Nanjing, China).

### Immunofluorescence staining

LF cells subjected to various treatments were fixed in 4% PFA for 30 min at room temperature. After permeabilizing and blocking treatment, the cells were incubated with primary antibodies against ɑ-SMA (1:1000, 55135-1-AP, Proteintech), Gli1 (1:1000, ab167388, Abcam) and (1:1000, 18166-AP, Proteintech) overnight at 4 °C, washed by PBS for three times and incubated with fluorescein isothiocyanate (FITC)-conjugated goat anti-rabbit antibody (1:1000, SA00001-2, Proteintech) in dark conditions. The nuclei were stained with DAPI (Beyotime, Nanjing, China) for 5 min. Immunofluorescence was observed under a fluorescence microscope (Olympus, Nanjing, China).

### Transfection

WISP-1 and Gli1 overexpression plasmids were provided by Nanjing Medical University (Nanjing, China). In brief, the human WISP-1 and Gli1 genes were subcloned into the lentiviral vector pLVX-IRES-ZsGreen1 (Siri, Nanjing, China). The WISP-1 and Gli1 overexpression plasmids were transfected into human LF fibroblasts with Lipofectamine 2000 (11668019, Thermo Fisher). After 24 h of transfection, cells were cultured in serum-free medium for another 24 h and then harvested for the subsequent experiment. Finally, the overexpression efficiency was tested by western blot analysis. For gene silencing, human WISP-1- and Gli1-specific shRNAs (Si-WISP1: 5-CCCAAGTACTGTGGAGTTT-3; shRNA: top strand: GATCCGCCCAAGTACTGTGGAGTTTTTCAAGAGAAAACTCCACAGTACTTGGGTTTTTTG, bottom strand: AATTCAAAAAACCCAAGTACTGTGGAGTTTTCTCTTGAAAAACTCCACAGTACTTGGGCG; Si-Gli1: 5-GGACAGAACTTTGATCCTT-3; shRNA: top strand: GATCCGGGACAGAACTTTGATCCTTTTCAAGAGAAAGGATCAAAGTTCTGTCCTTTTTTG, bottom strand: AATTCAAAAAAGGACAGAACTTTGATCCTTTCTCTTGAAAAGGATCAAAGTTCTGTCCCG) was cloned into the pHBLV-U6 vector (Siri, Nanjing, China) as described by the manufacturer. Lentiviruses containing the target gene shRNA were collected and used to transfect LF fibroblasts. The silencing efficiency of shRNA was also tested by western blot analysis.

### Mechanical stretch stimulation of LF fibroblasts

LF fibroblasts were seeded in a special chamber (Beyotime, Beijing, China) at 1 × 10^6^ cells per chamber. The chamber had a stretching apparatus in which cyclic stretching (5% and 10% elongation) was applied to the cells. In this study, 10% elongation stretching was applied for 6, 12, and 24 h (15 cycles/min, 37 °C, 5% CO_2_). After stretch stimulation, total RNA and protein were isolated to measure the expression of WISP-1.

### RT-PCR

According to the manufacturers’ instructions, total RNA was extracted from LF tissues and cells using TRIzol reagent (Invitrogen) and converted to cDNA using a Reverse Transcription Synthesis Kit (Takara, Dalian, China). Then, the gene levels were analyzed using a Thermal Cycler Dice Real-Time System (Takara, China). After the reaction, the threshold cycle (Ct) was determined, and the relative mRNA level was normalized to GAPDH expression using the 2^−∆Ct^ method. The designed primers and their sequences are summarized in Supplementary Table 1.

### Western blotting

Proteins from LF tissues and cells were extracted with a Protein Extraction Sample Kit (Sigma, USA). Subsequently, the proteins were separated by SDS-PAGE and transferred to nitrocellulose membranes (Sigma, USA) by electroblotting. The membranes were blocked for 2 h at room temperature with 5% skim milk (Sigma); probed with primary antibodies against WISP-1 (1:1000, 18166-AP, Proteintech), Smo (1:1000, 13576-AP, Proteintech), Gli1 (1:1000, ab167388, Abcam), Shh (1:1000, bs-1544R, Bioss), collagen I (1:1000, 14695-1-AP, Proteintech), collagen III (1:1000, bs-0549R, Bioss), Bax (1:1000, 50599-2-Ig, Proteintech), Bcl-2 (1:1000, 12789-1-AP, Proteintech), and α-SMA (1:1000, 55135-1-AP, Proteintech); and hybridized overnight at 4 °C with gentle shaking on a shaker. Finally, the membranes were incubated with goat anti-rabbit or anti-mouse secondary antibodies for 2 h at room temperature (1:1000, SA00001-2, Proteintech). GAPDH (1:1000, AP0063, Bioworld) was used as an internal control. The intensity of each blotting band was detected using a western blotting chemiluminescence kit (Bioworld).

### Rabbit LF degeneration and hypertrophy model

All surgical and experimental procedures were approved by the ethics committee of Nanjing Medical University. Nine female New Zealand white rabbits weighing ≈3.0 kg were randomly divided into three groups in this study. The rabbits were anesthetized using ketamine hydrochloride (30 mg/kg) and xylazine (10 mg/kg), and infection was prevented by injection of antibiotics (10 mg/kg cefazolin sodium pentahydrate; Shenzhen, China) via the ear vein. Then, the rabbits were placed in a prone position. A dorsal midline skin incision of approximately 6 cm was created under X-ray guidance. As described previously,^17^ the lumbosacral fascia to the left of the mammillary process was cut, and then the gap between the multifidus and longissimus muscles was located. Subsequently, we detached the multifidus from the mammillary processes to expose the lamina, the root of the transverse process and the posterior edge of the vertebral body. Group A underwent surgical exposure without fixation as a control (*n* = 3). Group B underwent posterolateral fusion with instrumentation (Watson locking plate; Changzhou, China) and underwent additional resection of the L3-4 supraspinal muscle and interspinous ligament to produce mechanical stress concentrated at the L3-4 level (*n* = 3). Briefly, a 4-hole titanium locking plate was used on the left posterolateral side of L2-3 and L4-5. A 2 mm × 10 mm titanium locking screw was then inserted into each vertebra and locked to produce mechanical stress concentrated at the L3-4 level. The rabbits in Group C were treated with cyclopamine (50 mg/kg every day by subcutaneous injection as described previously^18,19^ except that the same surgical procedure was performed as in Group B (*n* = 3)). All rabbits were raised independently with free access to food and water. The three groups of rabbits were sacrificed at 12 weeks after surgery.

### Statistical analysis

All the data are presented as the mean ± standard error of the mean. Statistical analyses were performed by using SPSS 17.0. The different groups were compared by using Student’s *t*-test. Correlations among LF thickness, fibrosis score and Gli1 expression were conducted by Pearson correlation analysis. We analyzed multiple groups using one-way or two-way ANOVA. *P* < 0.05 was considered to indicate statistical significance (*P < 0.05, ^$^*P* < 0.05, **P < 0.01, ^##^*P* < 0.01, ^$$^*P* < 0.01, ***P < 0.01).

## Results

### LF thickness and fibrosis score

Twenty-one patients with LSCS and 21 patients with LDH were recruited for the present study. No significant differences in age, sex, or the level of the LF samples were observed between the two groups (Table 1, *P* > 0.05).

In imaging analysis, the LF was found to play a dominating role in LSCS. The LF has been found to be 4–8 mm thick in patients with LSCS, while the thickness of the normal LF is 4 mm or less.^2,18^ Consistent with these results, the thickness of the LF was more than 4 mm in the LSCS group. This hypertrophic LF compressed the dural sac and nerve root and caused LSCS (Fig. 1a). In the LDH group, the LF thickness was less than 4 mm without compression (Fig. 1a). Briefly, the mean LF thickness in the LSCS group was 4.75 ± 0.39 mm, which was significantly higher than the 2.57 ± 0.69 mm in the LDH group (Table 1 and Fig. 1b). Moreover, based on MT staining, the fibrosis score of the dorsal side of the LF in the LSCS group was 3.14 ± 0.85, while that in the LDH group was 0.95 ± 0.92 (Fig. 1c, *P* < 0.05). Furthermore, correlation analysis demonstrated that the fibrosis score was positively correlated with LF thickness (*r* = 0.272, *P* < 0.05) (Fig. 1d), indicating that fibrosis was the main pathological feature of LF hypertrophy.

### Structural analysis of LF tissues

Growing evidence has demonstrated the structural disorder of LF tissues of LSCS patients.^2,5^ To fully reveal the structural changes in LF tissues, we next conducted immunohistochemical analysis and TEM to detect the basic structure and ultrastructure of the LF. As illustrated in Fig. 1e, the normal LF tissue from the LDH group was rich in elastic fibers. The fibers were arrayed in a regular order (H&E and EVG staining). Nevertheless, consistent with the findings of previous studies,^2,6^ the hypertrophic LF showed marked loss of elastic fibers in the LSCS group. According to morphological analysis, the elastic fibers were uneven, fragmented, disorganized, and partially absent. In MT staining, the elastic fibers were stained pink, while the collagen fibers were stained blue. In the LDH group, a large area was stained pink; however, in the LSCS group, most of the area was stained blue, showing fibrotic changes. Compared with the LDH group, the LSCS group exhibited a large number of fibrosis-related proteins, including collagen I and collagen III, as detected by immunohistochemistry.

Moreover, ultrastructural observation by TEM also showed that the LF tissues from the LDH group contained abundant elastic fibers and few collagen fibers (Fig. 1f). However, there was a significant loss of elastic fibers and an increase in the collagen fiber content in the LSCS group. The elastic fibers on the coronal plane in the LSCS group were mainly long and fusiform or irregular in shape, while those in the LDH group were mainly round or short and fusiform in shape (Fig. 1f).

### Hedgehog-Gli1 signaling is activated in hypertrophic LF tissue and highly associated with LF fibrosis

**Fig. 2:**
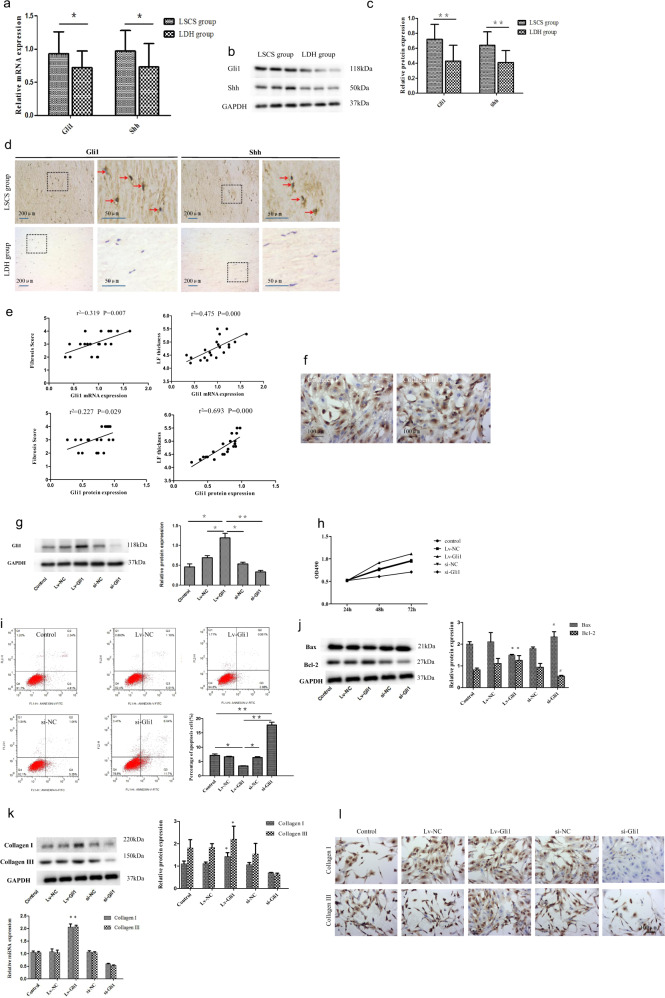
Hedgehog-Gli1 signaling is activated in hypertrophic LF and is important for cell activity and collagen expression. **a**–**c** Expression levels of Gli1 and Shh in LF tissues as detected by RT-PCR (**a**) and western blotting (**b**, **c**) (*n* = 21). **d** Representative images of immunohistochemical staining of Gli1 and Shh in LF specimens from the two groups (*n* = 3). **e** Correlation analysis of Gli1 expression with the fibrosis score and LF thickness. **f** Phenotype identification of LF cells; scale bar, 100 μm. **g** Gli1 overexpression plasmids and Gli1-silencing shRNAs were transfected into human LF fibroblasts, and cloning was performed. The efficiency of Gli1 expression was tested by western blotting. **h** MTT was used to detect the effect of Gli1 expression on LF cell proliferation. **i**, **j** The effect of Gli1 expression on LF cell apoptosis was assessed by flow cytometry (*n* = 3). **k**, **l** Effect of Gli1 on collagen expression in LF cells (*n* = 3); scale bar, 100 μm. The results shown are the means ± SDs; *P < 0.05, **P < 0.01, ^##^*P* < 0.001 compared with the control group. LF ligamentum flavum, LSCS lumbar spinal canal stenosis, LDH lumbar disc herniation.

To elucidate the molecular pathway involved in LF fibrosis, we focused on Hedgehog-Gli1 signaling, as this pathway was previously reported to be involved in fibrosis in various diseases.^20^ As expected, both the mRNA (Fig. 2a) and protein (Fig. 2b, c) levels of Gli1 and Shh were higher in LF samples from the LSCS group than in those from the LDH group (*P* < 0.05). In addition, we assessed the cellular sources of Gli1 and Shh in LF cells by immunohistochemical analysis of LF tissues. The number of cells positive for Gli1 and Shh was significantly greater in the LSCS group than in the LDH group (Fig. 2d). Moreover, the Gli1 level was positively correlated with the LF thickness (Fig. 2e, *P* < 0.05) and the fibrosis score (Fig. 2e, *P* < 0.05). Collectively, these data suggested that Shh signaling was activated in hypertrophic LF tissue.

### Effect of Hedgehog-Gli1 signaling on fibrogenesis in vitro

We next elucidated the mechanism by which activated Hedgehog-Gli1 signaling promoted fibrogenesis in vitro. It has been well demonstrated that the Hedgehog pathway modulates several important aspects of cell function, including cell proliferation, activation, and differentiation, and Hedgehog pathway targeting is a promising direction for fibrosis treatment.^21^ To further confirm the effect of Hedgehog-Gli1 signaling activation on LF cell viability, we analyzed the effect of Gli1 expression on the proliferation and apoptosis of LF cells. As shown in Fig. 2f, most of the cells were stained for collagen I or collagen III (each of which is a marker of LF fibroblasts), indicating the high purity of the LF fibroblasts obtained. Subsequently, we analyzed cell viability with or without Gli1 overexpression or knockdown (Fig. 2g) using an MTS assay and flow cytometry. As shown in Fig. 2h, cell proliferation was markedly promoted in Gli1-overexpressing LF fibroblasts compared with control fibroblasts, whereas the promoting effect was alleviated by knockdown of Gli1. Moreover, flow cytometry suggested that LF cells overexpressing Gli1 exhibited a much lower percentage of apoptosis than control cells (Fig. 2i). In contrast, the rate of apoptosis in Gli1 knockdown LF cells was higher than that in control cells (Fig. 2i). Further study revealed that the mechanism underlying the antiapoptotic effect in Gli1-overexpressing LF cells involved inhibition of Bax and activation of Bcl-2 (Fig. 2j). Together, the above data suggested that activated Hedgehog-Gli1 signaling enhanced fibroblast viability by promoting cell proliferation and inhibiting apoptosis.

To further determine the relationship between the activation of Hedgehog-Gli1 signaling and fibrogenesis in fibroblasts, we analyzed the effect of Gli1 overexpression or knockdown on the expression of fibrosis-related proteins such as collagen I and III in LF fibroblasts. As illustrated in Fig. 2j, overexpression of Gli1 significantly increased the expression of collagen I and III in LF cells. In contrast, the levels of collagen I and III in the Gli1 knockdown LF cells were lower than those in the control cells (Fig. 2k). Furthermore, immunohistochemistry demonstrated that overexpression or knockdown of Gli1 increased or abrogated the expression of fibrosis-related proteins, respectively (Fig. 2l), suggesting that activated Hedgehog-Gli1 signaling in LF cells promoted fibrogenesis in vitro. In this study, compared with Gli1 overexpression, Gli1 knockdown resulted in visually detectable decreases in the levels of collagen I and collagen III. This effect might have been related to the high interference efficiency of the siRNA.

### Mechanical stretch stress induces WISP-1 expression in LF cells

**Fig. 3:**
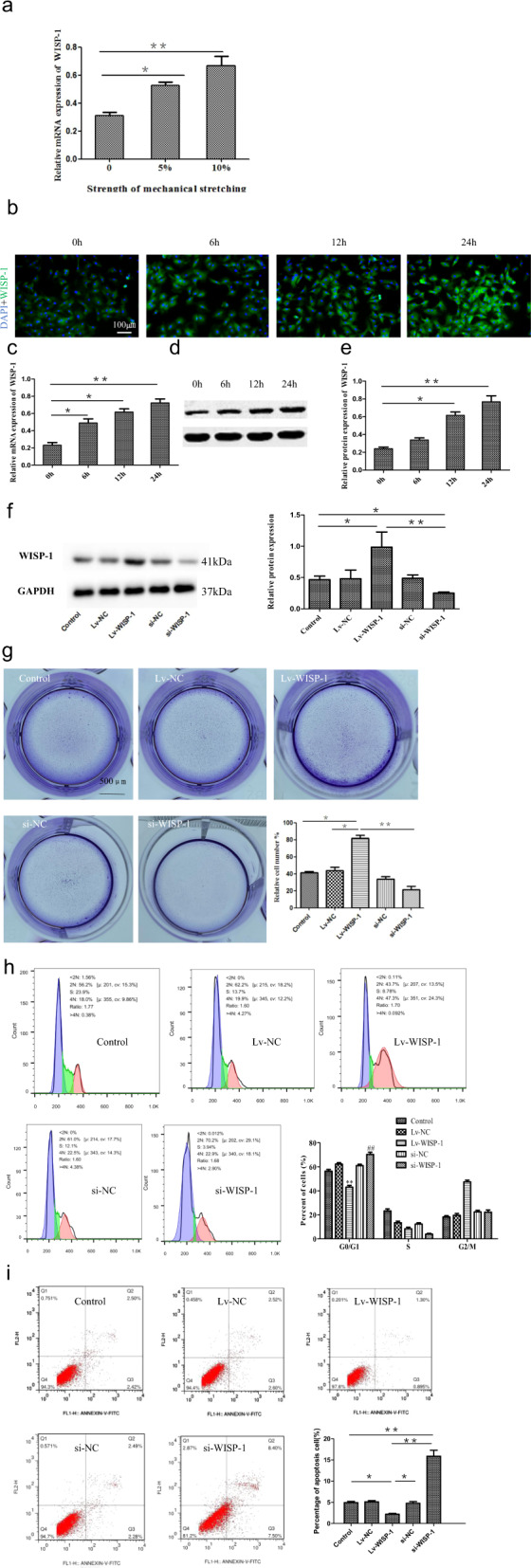
WISP-1 expression can be induced by mechanical stretch stimulation in fibroblasts, and WISP-1 promotes LF cell proliferation and inhibits apoptosis. **a** mRNA expression of WISP-1 under different grades of stretch stimulation. **b** Immunofluorescence staining of WISP-1 in LF cells at different time points under 10% elongation. Scale bar, 100 µm. **c**–**e** mRNA (**c**) and protein (**d**–**e**) expression of WISP-1 at different time points under 10% elongation (*n* = 3). **f** Protein expression of WISP-1 after different transfection protocols. **g** Colony-forming unit analysis demonstrated the effect of WISP-1 on LF cell proliferation. Scale bar, 500 µm. **h** Flow cytometry analysis of the effect of WISP-1 on the cell cycle of LF cells (*n* = 3). **i** The effect of WISP-1 on apoptosis was assessed by flow cytometry (*n* = 3). The results shown are the means ± SDs; *P < 0.05, **P < 0.01, ^##^*P* < 0.01 compared with the control group. LF ligamentum flavum.

Growing evidence has suggested that WISP-1 is involved in the process of fibrosis in various organs.^22,23^ Previously, we found that WISP-1 was markedly upregulated in hypertrophic LF tissue and strongly associated with several fibrosis-related factors, including collagen expression, LF thickness, and fibrosis scores. Mechanical stress is the main cause of LF degeneration.^24,25^ Therefore, we suspected that mechanical stress might have been the cause of the abnormal expression of WISP-1 in hypertrophic LF tissue. To test this possibility, we constructed an experimental device in which repeated cyclic mechanical stretch stimulation could be applied to LF fibroblasts obtained from patients with LSCS. As expected, WISP-1 mRNA expression in LF fibroblasts was markedly elevated by stretch stimulation, and the increase in WISP-1 expression tended to depend on the strength of the stretch stimulation (Fig. 3a). Furthermore, immunofluorescence analysis indicated that the expression of WISP-1 was increased by 6 h, 12 h, and 24 h of mechanical stretching (Fig. 3b). Consistently, the levels of WISP-1 mRNA (Fig. 3C) and protein (Fig. 3c–e) in LF cells were elevated in a time-dependent manner under the action of mechanical stretching (10% elongation).

### WISP-1 promotes the proliferation and inhibits apoptosis of LF fibroblasts

Previous studies have strongly implicated WISP-1 in the biological processes of fibroblast proliferation and migration.^22,23,26^ However, many questions remain about the precise ways in which WISP-1 regulates LF fibroblasts and subsequently regulates these biological processes. To answer these questions, we constructed LF cell lines with WISP-1 overexpression or knockdown (Fig. 3f). Colony formation assays revealed that overexpression of WISP-1 in LF cells significantly increased fibroblast proliferation. In contrast, WISP-1 knockdown inhibited the proliferation of LF fibroblasts (Fig. 3g). Furthermore, flow cytometry showed that WISP-1 promoted cell proliferation by inhibiting the G0/G1 phase transition of LF fibroblasts. Specifically, the percentage of LF cells in G0/G1 phase was higher in the group with WISP-1 silencing than in the control group. Moreover, the percentage of LF cells in G0/G1 phase was lower in the group with WISP-1 overexpression than in the control group (Fig. 3h). Moreover, flow cytometry analysis showed that WISP-1 inhibited LF cell apoptosis; the apoptosis rate of WISP-1-overexpressing LF cells was lower than that of control cells, whereas the apoptosis rate of cells with WISP-1 silencing was much higher than that of control cells (Fig. 3i). Overall, these data suggested that WISP-1 enhanced LF fibroblast viability by promoting cell proliferation and inhibiting apoptosis.

### WISP-1 activates Hedgehog-Gli1 signaling in LF fibroblasts

**Fig. 4:**
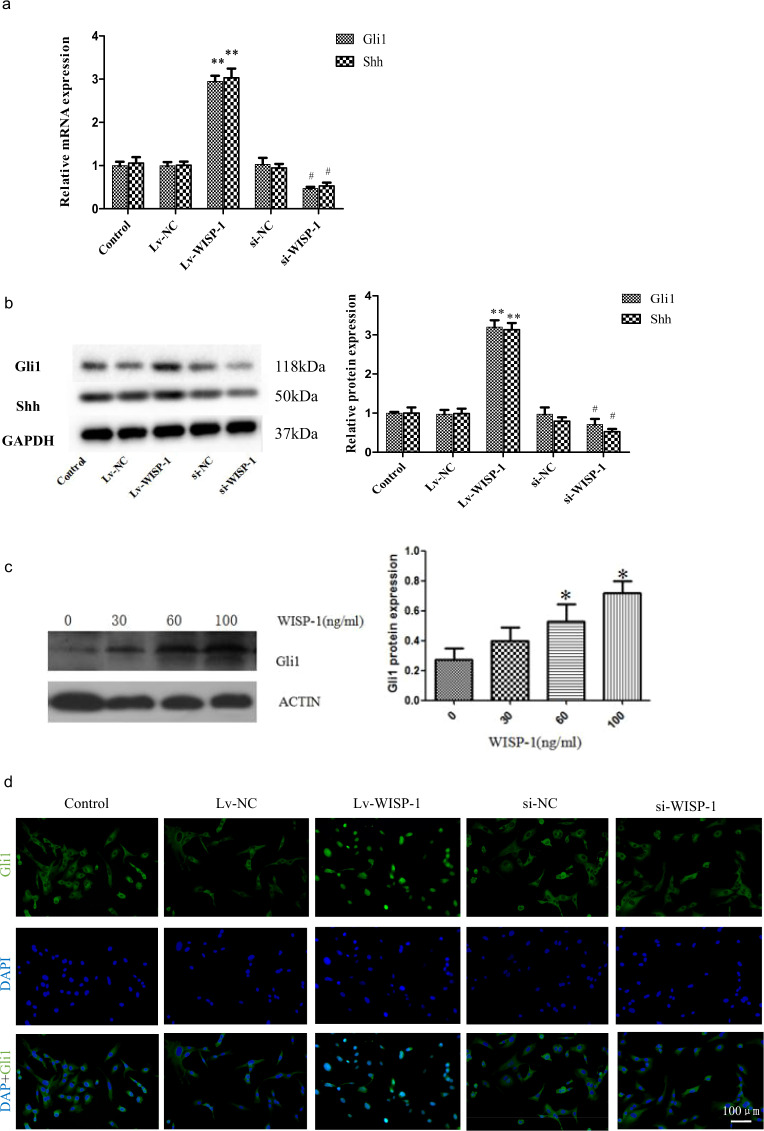
WISP-1 activates Hedgehog-Gli1 signaling in LF cells. **a**, **b** RT-PCR (**a**) and western blot (**b**) analyses of the effect of WISP-1 on the expression of Hedgehog-Gli1-related proteins in LF cells (*n* = 3). **c** Quantification of Gli1 protein expression in human LF cells treated with different doses of recombinant human WISP-1 (*n* = 3). **d** Effect of WISP-1 on the nuclear translocation of Gli1 in human LF cells as measured by immunofluorescence; scale bar, 100 μm. *P < 0.05, **P < 0.01 compared with the control group. LF ligamentum flavum.

To elucidate the underlying mechanism by which elevated WISP-1 promoted LF fibrosis, we analyzed the effects of WISP-1 overexpression and knockdown on the expression of Hedgehog signaling-related proteins, including Gli1 and Shh, in LF cells from LSCS patients. We found that overexpression of WISP-1 increased the levels of Gli1 and Shh in LF fibroblasts, while knockdown of WISP-1 inhibited the expression of Gli1 and Shh (Fig. 4a, b). Consistent with these results, recombinant human WISP-1 promoted Gli1 protein expression in a dose-dependent manner in WISP-1-treated LF cells (Fig. 4c).

Furthermore, we used immunofluorescence staining to investigate Gli1 expression and nuclear translocation upon WISP-1 treatment. The results revealed that Gli1 was preferentially distributed in the cytoplasm rather than in the nucleus in the control group. However, under WISP-1 overexpression, most Gli1 translocated to the nucleus. Moreover, Gli1 translocation was inhibited in WISP-1 knockdown LF cells (Fig. 4d), indicating that WISP-1 activated the Hedgehog-Gli1 pathway in LF fibroblasts.

### Cyclopamine suppresses WISP-1-induced fibrogenesis in vitro

**Fig. 5:**
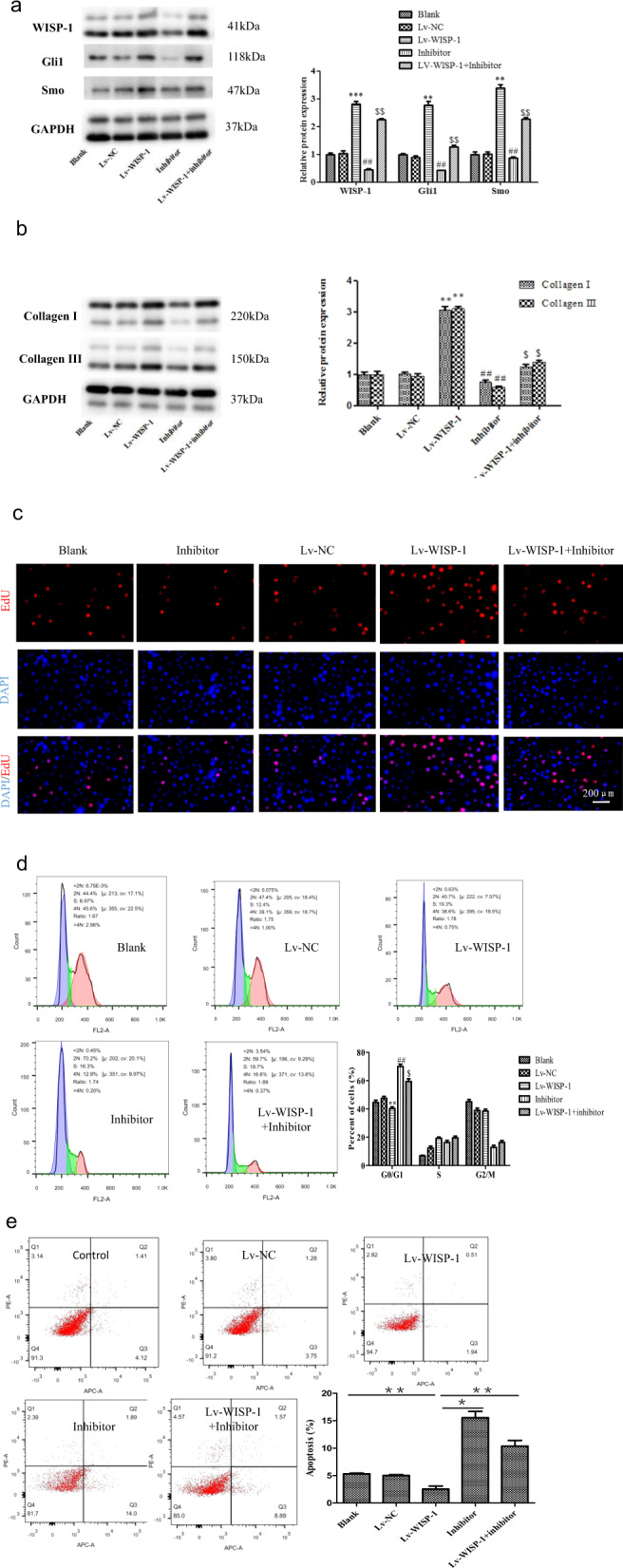
Cyclopamine suppresses WISP-1-induced fibrogenesis in fibroblasts. **a** Effect of WISP-1 on Gli1 and Smo expression in LF cells with or without cyclopamine (5 µM, *n* = 3). **b** Cyclopamine suppressed WISP-1-induced collagen expression (*n* = 3). **c** EdU staining demonstrated the effect of WISP-1 on LF cell proliferation with or without cyclopamine. Scale bar, 100 µm. **d** Effect of WISP-1 on the cell cycle of LF cells with or without cyclopamine (*n* = 3). **e** Flow cytometry was used to analyze the effect of Hedgehog signaling on WISP-1-induced apoptosis (*n* = 3). *P < 0.05, ^$^*P* < 0.05, **P < 0.01, ^##^*P* < 0.01 compared with the control group. LF ligamentum flavum.

As shown in Fig. 5a, WISP-1 expression increased after plasmid transfection, and the levels of Hedgehog signaling-related proteins such as Gli1 and Smo were significantly reduced by the Hedgehog signaling inhibitor cyclopamine. Previously, our in vitro experiment suggested that WISP-1 increased collagen expression in human LF fibroblasts.^14^ Thus, we sought to explore the intrinsic mechanisms by which WISP-1 regulated collagen expression. Based on the above data, we predicted that WISP-1 might increase collagen expression via the Hedgehog pathway. To confirm this prediction, we detected the effect of WISP-1 on the expression of collagen by adding a Hedgehog inhibitor (cyclopamine) to human LF cells. We found that the upregulation of collagen expression induced by WISP-1 was significantly attenuated by cyclopamine (Fig. 5b).

Having demonstrated that WISP-1 enhanced LF fibroblast viability, we next explored the possible mechanisms. According to the results of the EdU assay, LF cell proliferation was significantly enhanced by WISP-1 overexpression. However, cyclopamine inhibited WISP-1-induced cell proliferation, suggesting that WISP-1 promoted LF cell proliferation via Hedgehog signaling (Fig. 5c). Furthermore, flow cytometry demonstrated that the percentage of G0/G1-phase LF cells was significantly lower among WISP-1-overexpressing cells than among control cells. However, this difference was suppressed by incubation with cyclopamine (Fig. 5d).

Furthermore, we examined the percentage of apoptotic cells by flow cytometry. The sum of the percentages of early and late apoptotic cells was significantly reduced by WISP-1 overexpression in LF cells. However, this percentage was significantly increased after incubation with cyclopamine (Fig. 5e). Taken together, the above data suggested that WISP-1 promoted collagen expression and cell viability through the Hedgehog pathway.

### WISP-1 promotes α-SMA expression through Hedgehog signaling

**Fig. 6:**
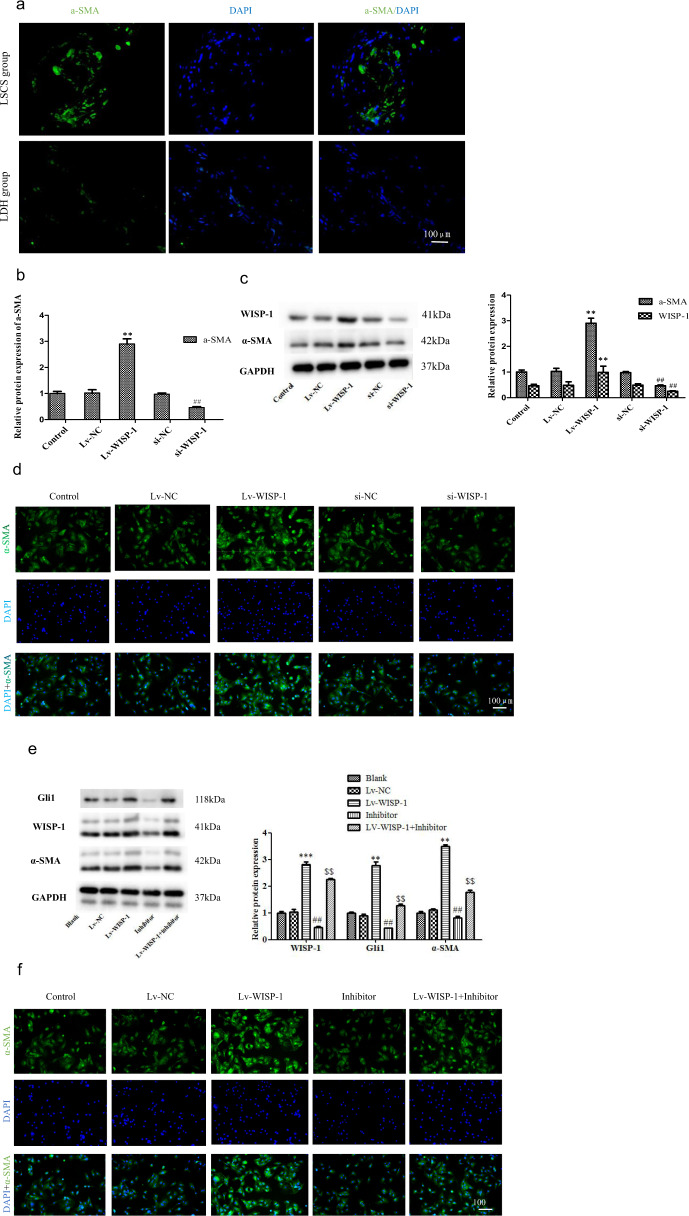
WISP-1 induces α-SMA expression via Hedgehog-Gli1 signaling. **a** Immunofluorescence analysis of α-SMA in human LF specimens. **b**–**d** Effect of WISP-1 on α-SMA expression in human LF cells as detected by RT-PCT (**b**), western blotting (**c**) and immunofluorescence (**d**) (*n* = 3). **e**, **f** Effect of cyclopamine on WISP-1-induced α-SMA expression as measured by western blot (**e**) and immunofluorescence staining (**f**) (*n* = 3). Scale bar, 100 µm. **P < 0.01, ^$$^*P* < 0.05, ^##^*P* < 0.01, ***P < 0.001, compared with the control group. LF ligamentum flavum, LSCS lumbar spinal canal stenosis, LDH lumbar disc herniation.

During fibrosis, the transdifferentiation of fibroblasts into myofibroblasts influences the production of ECM and the secretion of fibrosis-related factors, which is key cellular events that drives the fibrosis response in various tissues and organs. α-SMA is regarded as a marker of activated myofibroblasts.^27–29^ We found that the fluorescence intensity of α-SMA in LF tissues from the LSCS group was significantly higher than that in LF tissues from the LDH group (Fig. 6a), indicating that the transition of fibroblasts into myofibroblasts was also an important mechanism of LF fibrosis. It has been reported that the CCN protein induces α-SMA expression during fibrosis in many organs.^30,31^ As expected, we also found that WISP-1 overexpression significantly increased α-SMA expression. In addition, the crucial role of WISP-1 in α-SMA expression in LF cells was further demonstrated by the loss-of-function experiment in which we knocked down WISP-1. The results showed that WISP-1 knockdown significantly reduced α-SMA expression (Fig. 6b, c). Further evidence of the role of WISP-1 in α-SMA expression was obtained via cell immunofluorescence measurement (Fig. 6d).

As we found that Hedgehog-Gli1 signaling was required for WISP-1-induced fibrogenesis, we predicted that WISP-1 might also increase α-SMA expression via the Hedgehog-Gli1 pathway. As expected, western blot analysis revealed that the protein expression of α-SMA in the LF cells was significantly reduced by cyclopamine (Fig. 6e). Consistent with this result, immunofluorescence analysis demonstrated that the increase in the fluorescence intensity of α-SMA induced by WISP-1 was significantly suppressed by cyclopamine (Fig. 6f). These results indicated that WISP-1 induced α-SMA expression in human LF fibroblasts by targeting Hedgehog signaling.

### Cyclopamine prevents mechanical stress-induced LF fibrosis and hypertrophy in vivo

**Fig. 7:**
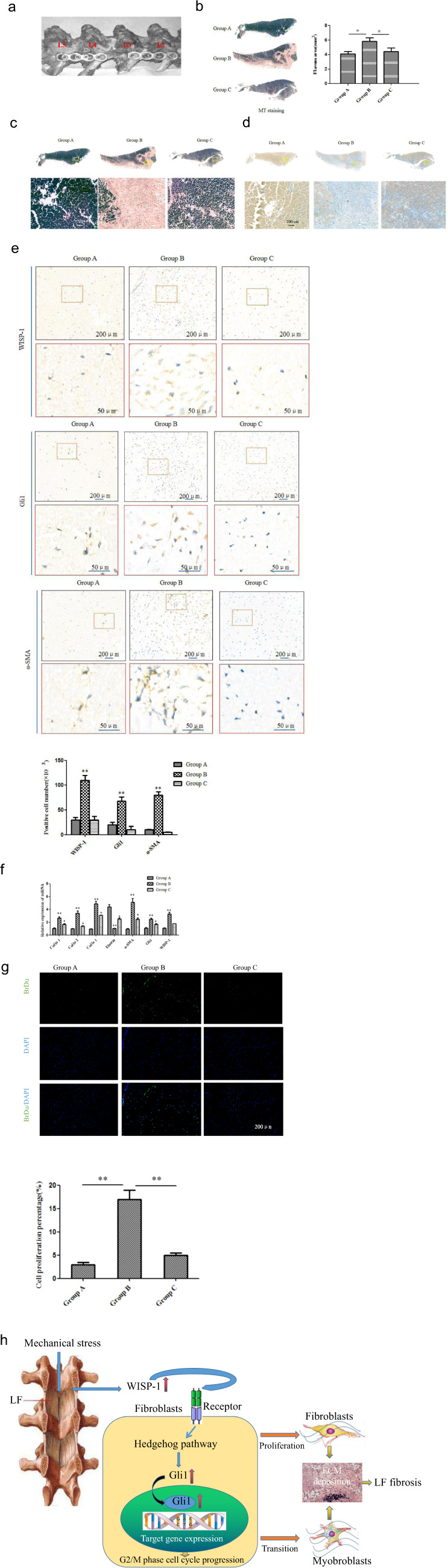
Cyclopamine prevents mechanical stress-induced LF fibrosis and hypertrophy in vivo. **a** Lateral view of the rabbit model with L2-3 and L4-5 spinal fusion. **b** The thickness of the LF was assessed to compare the area of the LF in each specimen with MT staining at the same level. **c**, **d** Representative images of EVG and MT staining of the LF. Scale bar, 200 µm. **e** Expression of WISP-1, Gli1, and a-SMA as detected by immunohistochemical analysis in the three groups and quantified using Image-Pro Plus 6.0. **f** mRNA expression profile of the LF. **g** Comparison of the number of proliferating cells (BrdU, green; DAPI, blue) in the three groups. Scale bar, 200 µm. (*P < 0.05: Group B compared with Group A; **P < 0.01: Group C compared with Group B). **h** Proposed model for the mechanism by which WISP-1/Hedgehog-Gli1 signaling participates in LF fibrosis. In response to mechanical stress, upregulated WISP-1 activates Hedgehog-Gli1 signaling in fibroblasts, which results in the posttranscriptional expression and nuclear translocation of Gli1, promotes cell proliferation and the fibroblast–myofibroblast transition, and inhibits apoptosis, ultimately leading to collagen deposition and LF fibrosis. (*P < 0.05, **P < 0.01; *n* = 3 for each group. Group A: control group; Group B: underwent posterolateral fusion to produce mechanical stress concentrated at the L3-4 level; Group C: treated with cyclopamine and subjected to the same surgical procedure used for Group B).

To further confirm the role of the WISP-1/Hedgehog pathway in LF, we built a rabbit model in which mechanical stress was concentrated at the level of the L3-4 segment with fixation of adjacent segments (L2-3 and L4-5, Fig. 7a). The results showed that the L3-4-level LF in Group B was thicker than that in group A (Fig. 7b). In addition, the structure of hypertrophic LF in rabbits was similar to that in humans (Fig. 7c, d).^17^ These results demonstrated that degeneration and hypertrophy of the LF occurred under continuous mechanical stress. Briefly, the L3-4-level LF in Group B showed a significant decrease in the density of elastic fibers and an increase in the collagen fiber content (Fig. 7c, d). The mRNA levels of fibrosis-related genes in the L3-4-level LF, including WISP-1, Gli1, Col1a2, Col2a1, and Col3a1, were significantly elevated in Group B compared with Group A, whereas the mRNA level of elastin was significantly decreased (*p* < 0.01) (Fig. 7e, f). These data indicated the activation and critical role of WISP-1/Hedgehog-Gli1 signaling in LF with hypertrophy induced by mechanical stress in a rabbit model and were consistent with the results observed in hypertrophic LF tissue in humans.

The in vivo effect of cyclopamine was also analyzed in the rabbit LF hypertrophy model. The results showed that LF thickness was significantly lower in the cyclopamine-treated group (Group C) than in Group B, whereas Group C showed approximately the same thickness as Group A (Fig. 7b). Cyclopamine treatment of the LF in Group C attenuated the upregulation of fibrosis-related genes such as collagen, Gli1 and a-SMA and the downregulation of elastin compared to the levels in Group B (Fig. 7e, f). In addition to these changes in ECM components, we detected cellular changes in rabbit LF samples. The number of BrdU-positive proliferating cells was significantly higher in Group B than in Group A, whereas the increase was reduced by cyclopamine treatment in Group C (Fig. 7g).

## Discussion

In this study, we confirmed, for the first time, that mechanical stress induces WISP-1 expression in LF cells obtained from LF tissues of LSCS patients. Moreover, we demonstrated that WISP-1 induces LF hypertrophy and fibrosis by activating the Hedgehog-Gli1 pathway and aggravating ECM deposition by promoting LF cell proliferation, inhibiting apoptosis and inducing the transition of fibroblasts into myofibroblasts. Most importantly, we found that inhibition of Hedgehog signaling suppresses mechanical stress-induced LF fibrosis in a rabbit model, indicating that targeting Hedgehog signaling may be a novel strategy for the prevention and treatment of LF fibrosis.

LSCS is a very common disease in the elderly population and threatens human health and life.^1,32,33^ Currently, LF hypertrophy is considered to be a major pathogenic factor in the development of LSCS.^2^ Consistent with this viewpoint, the present study demonstrated that the LF in the LSCS group was significantly thicker than that in the LDH group. Histologically, the hypertrophic LF showed a decrease in elastic fiber content and accumulation of collagen, suggesting the occurrence of fibrotic changes consistent with those reported by previous studies.^11,33^

Emerging evidence has suggested that WISP-1 is involved in the initiation and progression of fibrosis in various organs.^13,18,19^ Our previous studies have shown that WISP-1 expression is increased in hypertrophic LF tissue and is strongly associated with LF fibrosis.^14^ However, the upstream mechanism of the abnormal expression of WISP-1 in LF tissue has remained unclear. Of note, it has been suggested that excessive mechanical stress commonly contributes to various pathological diseases.^34,35^ Such mechanical stress owing to segmental instability usually occurs in patients with LSCS, and the increasing instability is considered to play a vital role in the development of LF fibrosis, ultimately leading to LF hypertrophy.^24,25,36^ Histologically, the degeneration of elastic fibers accelerates, and the accumulation of collagen markedly increases because of mechanical stress.^37^ Therefore, we were interested in examining whether mechanical stress induces WISP-1 expression during fibrogenesis. As expected, we found that mechanical stretch stress directly induced WISP-1 expression in vitro and in vivo.

Subsequently, we investigated the potential role of WISP-1 in LF fibrosis and the probable molecular mechanism. As previously reported, Hedgehog signaling is a critical mechanism underlying persistent fibroblast activation in fibrotic diseases.^38–40^ The Gli family members are downstream transcription factors of the Hedgehog pathway.^41^ Notably, Gli1 is the main transcription factor of Hedgehog signaling and can be viewed as a readout of pathway activity.^42^ Generally, Gli1 is present in the cytoplasm. Once activated, Gli1 is cleaved and transported to the nucleus, resulting in transcription of Hedgehog genes.^43^ Consistent with this mechanism, our results revealed that Hedgehog signaling was activated during LF fibrosis. Furthermore, we found that WISP-1 activated the Hedgehog-Gli1 pathway in LF cells. Notably, growing evidence suggests that cyclopamine plays a crucial role in treating liver fibrosis by inhibiting several fibrotic genes.^44,45^ Similarly, we demonstrated that cyclopamine inhibited WISP-1-induced fibrogenesis in vitro and in vivo, suggesting that Hedgehog-Gli1 signaling is one of the targets of WISP-1 and mediates the fibrotic effect of WISP-1.

Myofibroblasts produce numerous ECM adhesive and structural proteins and are characterized by the expression of α-SMA.^46–48^ The transdifferentiation of fibroblasts into myofibroblasts is a key cellular event driving the fibrosis response in various tissues and organs.^24,25,36^ It has been reported that activated Hedgehog signaling promotes the fibroblast-to-myofibroblast transition and fibrosis, whereas inhibition of this signaling ameliorates fibrosis in several diseases.^38–40^ We demonstrated that α-SMA was upregulated in hypertrophic LF tissues, indicating that the fibroblast-to-myofibroblast transition is an important mechanism of LF fibrosis. Furthermore, we found that WISP-1 promoted α-SMA expression through Hedgehog signaling, indicating that mechanical stress/WISP-1/Hedgehog/α-SMA signaling is a new profibrotic axis.

It is generally accepted that integrins are key cell surface receptors for the CCN family.^22,23,26^ Our ongoing study seeks to identify the corresponding receptors that are responsible for determining the selective effects of WISP-1 on the Hedgehog pathway. Overall, the present data reveal the important role of the mechanical stress/WISP-1/Hedgehog axis in LF fibrosis and contribute to a better understanding of LF hypertrophy (Fig. 7h). The results from our in vitro and in vivo experiments indicate that interfering with Hedgehog signaling may be a novel strategy for the prevention and treatment of LF fibrosis and hypertrophy.

## Supplementary information

supplementary table 1

## Data Availability

The data that support the findings of this study are available from the corresponding author upon reasonable request.
