# Supplementary material for: WISP-1 induced by mechanical stress contributes to fibrosis and hypertrophy of the ligamentum flavum through Hedgehog-Gli1 signaling
Source: Exp Mol Med. 2021 Jun 22;53(6):1068–79. doi: 10.1038/s12276-021-00636-5 (PMC8257797; doi:10.1038/s12276-021-00636-5)
Supplement: Supplementary file 1 — supplementary table 1 [file 12276_2021_636_MOESM1_ESM.doc]

Supplementary Table 1 RT-PCR primers used in this study（human and rabbit）

| Primer | Race | Sequence | Size(bp) | Annealing temperature(℃) |
| --- | --- | --- | --- | --- |
| Gli1 | human | Forward: 5′-TTCCTACCAGAGTCCCAAGT-3′  Reverse: 5′ -CCCTATGTGAAGCCCTATTT-3′ | 118 | 60 |
|  | rabbit | Forward: 5′ -CTGCTGTGGGAGGGTGGG-3′  Reverse: 5′ -CCTTGTGATTGGGCAAACTGG-3′ | 159 | 60 |
| Shh | human | Forward: 5′-GAGCGGACAGGCTGATGACT-3′  Reverse: 5′ -GCTTTCACCGAGCAGTGGATA-3′ | 50 | 60 |
| WISP-1 | human | Forward: 5′-CCTGCGACCCTGTGATGTGG-3′  Reverse: 5′ -CACGCTGATGGTCTTGGACTTGTAG-3′ | 41 | 60 |
|  | rabbit | Forward: 5′ -CCTGCGACCCTGTGATGTGG-3′  Reverse: 5′ -CACGCTGATGGTCTTGGACTTGTAG-3′ | 195 | 60 |
| α-SMA | human | Forward: 5′ -CATTTGGTCAGAAGACGGTTG-3′  Reverse: 5′ -GACCTGGAGTTCTCACTTTCATC-3′ | 42 | 60 |
|  | rabbit | Forward: 5′ -CCCCTGAAGAACATCCAACC-3′  Reverse: 5′ -CCATCTCCAGAGTCCAGCACA-3′ | 183 | 60 |
| Collagen I | human | Forward: 5′ -ATGCCTGGTGAACGTGGT-3′  Reverse: 5′ -AGGAGAGCCATCAGCACCT-3′ | 220 | 60 |
| Collagen III | human | Forward: 5′ -CGCTCTGCTTCATCCCACTATTA-3′  Reverse: 5′ -ATTTGGCATGGTTCTGGCTTC-3′ | 150 | 60 |
| GAPDH | human | Forward: 5′ -AGAAGGTGGTGAAGCAGGCGTC-3′  Reverse: 5′ -AAAGGTGGAGGAGTGGGTGTCG-3′ | 37 | 60 |
|  | rabbit | Forward: 5′ -CGCCTGGAGAAAGCTGCTAAG-3′  Reverse: 5′ -GACGACCTGGTCCTCGGTGTAG-3′ | 104 | 60 |
| Elastin | rabbit | Forward: 5′ -GCCTGGGCTTGGAGTTGGTGC-3′  Reverse: 5′ -CACGCCTCCCGCTCCGTATTTC-3′ | 156 | 60 |
| Col1a1 | rabbit | Forward: 5′ -CAAGACCACCAAGACCTCCCG-3′  Reverse: 5′ -GTCTGGGTTGTTTGTCGTCTGTTTC-3′ | 191 | 60 |
| Col1a2 | rabbit | Forward: 5′ -GCGGTGGTTACGACTTTGG-3′  Reverse: 5′ -CCTTCAGGAGTGAGGAGGGTCT-3′ | 147 | 60 |
| Col3a1 | rabbit | Forward: 5′ -CGAGCCTCCCAGAACATCAC-3′  Reverse: 5′ -GAGCAGCCATCCTCCAGAAC-3′ | 166 | 60 |
